# Trends in *Arthrospira* sp. (Spirulina) Applications: A 15-Year Bibliometric Analysis and Systematic Review

**DOI:** 10.3390/plants15060857

**Published:** 2026-03-10

**Authors:** Hoda Hosseini, Touria Bounnit, Imen Saadaoui

**Affiliations:** 1 Biotechnology Program, Centre for Sustainable Development, College of Arts and Sciences, Qatar University, Doha P.O. Box 2713, Qatar; 2 Algal Technology Program, Centre for Sustainable Development, College of Arts and Sciences, Qatar University, Doha P.O. Box 2713, Qatar; touria.bounnit@qu.edu.qa

**Keywords:** *Arthrospira* sp., biotechnological applications, Phycocyanin, market value

## Abstract

Due to their exceptional nutritional benefits, microalgae and cyanobacteria are recognized as sustainable food sources and key contributors to the circular bioeconomy. *Arthrospira* sp. has garnered significant attention as one of the most promising cyanobacteria for a wide range of applications. The purpose of this study is to systematically analyze and synthesize global research trends in *Arthrospira* sp. applications. In this context, a “systematic review” refers to an integrated bibliometric and thematic analysis encompassing publication trends, geographical distribution of research outputs, leading journals, key application sectors, market development, and associated challenges and future prospects. Consequently, extensive research has been conducted on this species, leading to diverse areas of interest and application. This review article is the first of its kind, offering a comprehensive summary of trends in *Arthrospira* sp. applications over the past 15 years. It presents a bibliometric analysis of publications from 2010 to 2024 in journals indexed by Scopus. The analysis revealed that Bioresource Technology is the leading journal in publishing related research, with China producing the highest number of studies. Furthermore, phycocyanin extraction emerged as the most frequently studied application. Recently explored applications include its use as a biofertilizer, in bioplastic production, and in cosmetics. The *Arthrospira* sp. market is currently valued at an estimated $619 million in 2024, positioning it as a dominant player in the global industry. However, challenges persist, including safety concerns related to potential allergies and toxicity, as well as regulatory hurdles that may affect commercialization and market expansion.

## 1. Introduction

The United Nations projects that the global population will reach approximately 9.7 billion by 2050, representing a 19% increase from today’s population [1]. This rapid demographic growth, together with ongoing urbanisation, industrial expansion, and rising per capita consumption, will place unprecedented demands on global food production systems. Meeting these demands poses substantial challenges, including risks to food security, environmental degradation, and increasing pressure on conventional agricultural practices [2]. In this context, there is a pressing need to complement traditional food supply systems with more sustainable, efficient, and resilient approaches, among which algaculture has emerged as a highly promising solution.

Microalgae offer exceptional potential due to their remarkable biomass productivity, adaptability to diverse environmental conditions, and rich nutritional profile [3]. Unlike conventional crops, they can be cultivated on non-arable land and in a wide range of water sources—including brackish water and seawater—thereby minimising competition with freshwater resources and fertile soil. Their cultivation can also leverage organic waste streams, nutrients from wastewater, or byproducts from other industrial processes, aligning closely with the principles of a circular economy and sustainable resource management. Beyond their environmental advantages, microalgae can be tailored to produce specific bioactive compounds, such as proteins, lipids, carbohydrates, and pigment profile [4] with diverse applications across the food, feed, and aquafeed [5,6], nutraceutical, pharmaceutical [7], cosmetics, bioplastics, biofertilizer, energy, and wastewater treatment sectors [8], in addition to their primary role in carbon dioxide mitigation [9].

This combination of high productivity, multifunctionality, and environmental adaptability underscores the strategic importance of microalgae in advancing sustainable food systems, bio-based industries, and climate mitigation initiatives. Furthermore, the bio-based compounds derived from microalgae are typically biodegradable and environmentally friendly, reducing ecological impact throughout their lifecycle, from production to consumption and disposal [10].

Reflecting this growing interest, the global microalgae market was valued at approximately USD 1 billion in 2022 and is projected to grow to USD 1.6 billion by 2030 (CAGR ~5.7%), with broader forecasts estimating up to USD 3.08 billion by 2030, depending on scope and included applications [11]. The majority of this growth is driven by applications in the food and health sectors, supported by consumer demand for sustainable, nutrient-dense, and functional products.

Among the industrially exploited algae strains, *Arthrospira* sp., commonly known as Spirulina, has received particular attention due to its commercial viability, nutritional value, and multifunctional applications. The significance of *Arthrospira* sp. is not only contemporary but also historical: it is recorded that the Aztecs systematically consumed *Arthrospira* sp. harvested from Lake Texcoco as a nutritious food source centuries ago, highlighting its long-standing human use and dietary importance [12]. Global production of *Arthrospira* sp. is estimated at approximately 12,000 tons of dry biomass annually [10], making it one of the most widely cultivated and economically significant cyanobacteria. Its high protein content—up to 70% of dry weight—positions it as one of the most protein-rich foods available. In addition to its nutritional profile, *Arthrospira* sp. exhibits potent bioactive properties, including antioxidant, anti-inflammatory, and immune-modulatory effects, which have contributed to its growing popularity as a functional food.

The safety and nutritional benefits of *Arthrospira* sp. are well-documented. Regulatory agencies, including the U.S. Food and Drug Administration (FDA), have classified it as “Generally Recognised as Safe” (GRAS), and the World Health Organisation (WHO) has acknowledged it as a superfood [13]. This regulatory recognition, coupled with rising consumer demand for natural, sustainable, and health-promoting products, has further stimulated its global market expansion, reinforcing its pivotal role in the emerging bioeconomy.

This article provides a comprehensive and up-to-date overview of research trends in *Arthrospira* sp. applications over the past fifteen years, addressing the current lack of an integrated analysis that simultaneously captures scientific, technological, and industrial developments in this field. Existing reviews have primarily focused on specific aspects such as nutritional value and health benefits [14,15,16], phycocyanin extraction and downstream processing [17,18,19], or cultivation strategies and sustainability considerations [20,21,22]. However, a consolidated assessment that combines long-term publication trends, application evolution, geographical research distribution, leading journals, and industrial and market perspectives of *Arthrospira* sp. remains limited.

To bridge this gap, the present study systematically examines the geographical distribution of research contributions, identifies the most influential journals in the field, and evaluates the major commercial players driving industrial-scale adoption of *Arthrospira* sp. Furthermore, it discusses current challenges, including safety concerns and regulatory constraints, and outlines prospects for the sustainable production and application of *Arthrospira* sp. within the framework of the circular bioeconomy. By doing so, this review highlights the potential role of *Arthrospira* sp. in supporting environmentally sustainable practices and contributing to global food security.

## 2. Results and Discussion

### 2.1. Descriptive Analysis of the Bibliometric Data

#### 2.1.1. Types of Retrieved Documents

**Figure 1:**
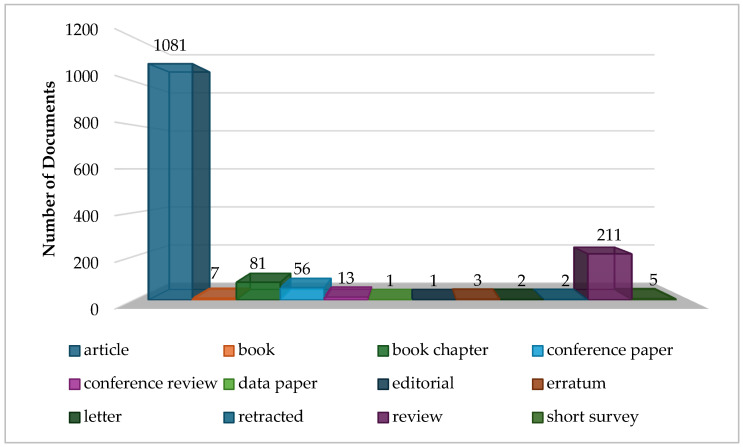
Number of documents resulting from the search based on type.

A Scopus database search yielded a total of 1463 documents related to *Arthrospira* sp. and its diverse applications, as illustrated in Figure 1. The distribution of these publications according to document type reveals that research articles dominate the field, accounting for over 75% of the total (1081 documents). This predominance reflects the substantial experimental and applied research efforts undertaken to explore the nutritional, functional, and industrial potential of *Arthrospira* sp. Following research articles, review articles constitute the second-largest category, with 211 documents, highlighting a significant interest in synthesising existing knowledge, identifying trends, and proposing future research directions. reflecting a growing scholarly interest in synthesising current knowledge, identifying emerging trends, and providing guidance for future research directions. Book chapters, representing 81 documents, offer in-depth discussions, case studies, and theoretical frameworks that complement the findings reported in primary research. This distribution of document types underscores a mature yet rapidly evolving research landscape, where primary research and knowledge synthesis complement each other to advance both scientific understanding and practical utilisation of *Arthrospira* sp.

Distribution of document types in the Spirulina-related literature included in the bibliometric analysis. The dataset is dominated by research articles (1081) and review papers (211), followed by book chapters, conference papers, and other publication types.

#### 2.1.2. Production Rate of Documents Related to *Arthrospira* sp.’s Applications

**Figure 2:**
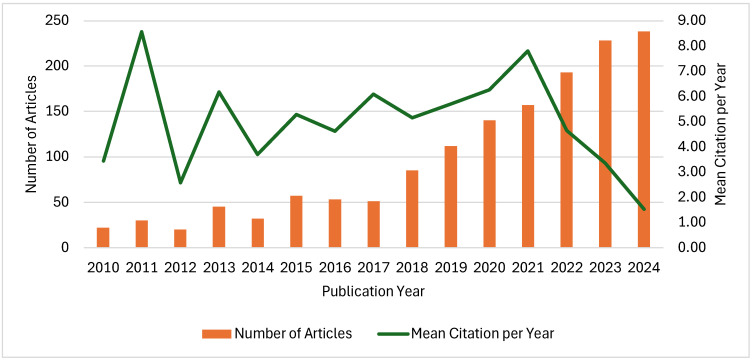
Global Annual documents’ production and citations.

The production of scientific literature on *Arthrospira* sp. applications has shown a marked upward trend over the period from 2010 to 2024, as represented in Figure 2. During this period, the total number of publications grew steadily, reflecting the increasing global research interest in the potential applications of this cyanobacterium. Correspondingly, the number of citations has also risen significantly, from 22 citations in 2010 to 238 in 2024, indicating that the research is not only expanding in volume but also gaining visibility and influence within the scientific community.

Despite the overall increase in publication numbers, the mean citations per document have shown fluctuations over the years (Figure 2). One important factor underlying this trend is the citation lag or citation window effect, a well-recognised bibliometric phenomenon whereby newly published articles require time before accruing citations. In bibliometric analyses, older publications typically have higher citation counts because they have had more time to be cited, whereas recent publications have a shorter window to attract citations, resulting in temporarily lower citation counts for the most recent years. This time-dependent accumulation of citations has been documented in the literature, where citation windows of at least 2–3 years are often needed for citations to stabilise and provide reliable impact measures, and newer articles may not yet have had sufficient time to be referenced by subsequent research [23].

Additionally, differences in the impact and visibility of studies across subfields can contribute to variability in citation counts, with some articles rapidly gaining attention while others integrate more slowly into the research landscape. Together, these factors explain why, despite the rapid growth in *Arthrospira* sp. publications, the average citations per document appear to decrease in more recent years.

#### 2.1.3. Most Important Sources of Documents

**Figure 3:**
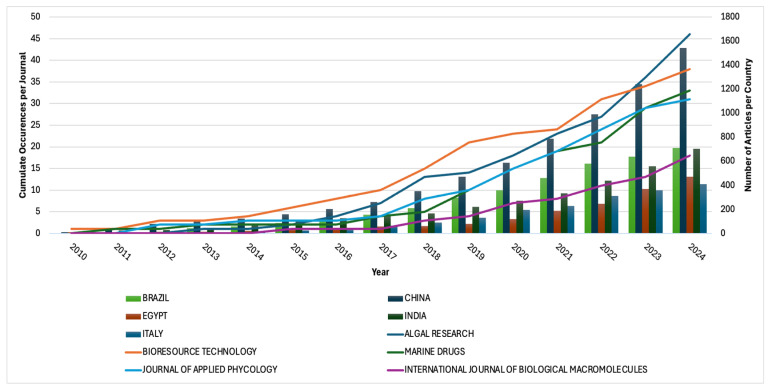
Annual production of documents based on journals.

The sources of publications on *Arthrospira* sp. applications were analysed based on both journals and countries of origin, as illustrated in Figure 3. Among the journals, Bioresource Technology emerged as the leading publisher of research articles in this field, reflecting its strong focus on biotechnological and applied research. Algal Research also demonstrated significant contributions, particularly in 2024, when it recorded a notable increase in published articles. Other prominent journals include Applied Phycology, Marine Drugs, and the International Journal of Biological Macromolecules, all of which consistently disseminate research on the nutritional, pharmaceutical, and industrial applications of *Arthrospira* sp.

Analysis of the country of origin of these publications reveals a relatively stable contribution pattern over the years. In 2024, China led the global output with 1543 publications, approximately double the number produced by Brazil and India, highlighting its dominant role in *Arthrospira* sp. research. The significance of *Arthrospira* sp. in China is both historical and industrial: while historically microalgae-based foods have been consumed in regions with alkaline lakes suitable for natural growth, contemporary China has developed large-scale cultivation and industrial applications. China’s leadership can be attributed to substantial national investments in microalgae research, numerous dedicated research institutions, industrial-scale Spirulina production facilities, and strong government support for sustainable food, biotechnology, and bio-based industries. For example, one of the largest providers of Spirulina in the world with 1.000 t year^−1^ with 400.000 m^2^ greenhouses is Yunnan Spirin Co., Ltd. which was established in China in 1992 [24] and today Dainippon Ink Corporation (DIC) is one of the largest Spirulina producers in the world, owning a production company in Hainan, China and Earthrise Nutritionals LLC [25]. Cultural and dietary acceptance of microalgae-based foods, along with government support for innovation in nutrition and environmental technologies, has also contributed to a high publication output. Brazil and India primarily focus on food, feed, and nutraceutical applications, reflecting their established aquaculture and livestock sectors. Egypt has emphasised cultivation optimisation, bioactive compound extraction, and local industrial applications, while Italy shows significant interest in nutraceutical and cosmetic applications [26], often linked to small and medium-sized enterprises in the bioeconomy sector. This distribution underscores the global interest in *Arthrospira* sp., while also illustrating that research output is concentrated in countries with strategic priorities, established infrastructure, and active industrial engagement in biotechnology and circular bioeconomy development.

### 2.2. Trends and Dynamics of Arthrospira sp.’s Applications

#### 2.2.1. Hotspots in Research Involving *Arthrospira* sp.’s Applications

The increasing global demand for plant-based and sustainable products has propelled *Arthrospira* sp. into the scientific and commercial spotlight. In recent years, its applications have expanded markedly, fueled by growing consumer awareness of health, wellness, and environmentally friendly solutions. Its multifunctional potential has positioned it as a versatile resource across multiple sectors, from nutrition and therapeutics to environmental management.

**Figure 4:**
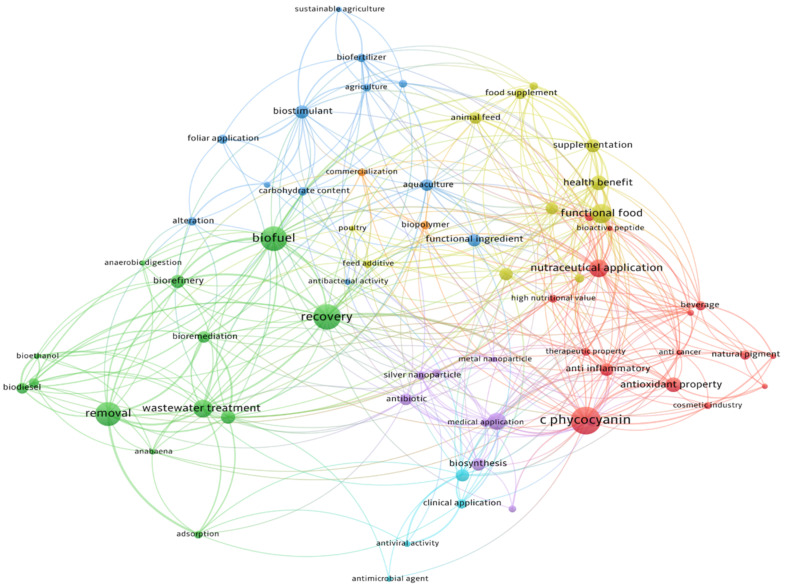
Overview of the different *Arthrospira* sp. applications and related research focal points using Keyword co-occurrence network analysis.

A keyword co-occurrence network analysis (Figure 4) provides a clear overview of the main research domains for *Arthrospira* sp. applications, which can be categorised into four primary clusters highlighting the diverse and interdisciplinary nature of its utilisation: therapeutic applications, waste treatment, foliar applications, and food and feed applications. Among these, the extraction and utilisation of phycocyanin emerge as the most prominent focus. This vibrant blue pigment accounts for up to 20% of its dry weight and presents several potential applications. Phycocyanin is primarily used as a natural food colouring with a price of $500/kg [27,28]. Commercially, it is marketed under brand names such as “Lina Blue” by Dainippon Ink, and is also extracted by the French company Algosource, which cultivates *Spirulina* and markets phycocyanin-rich extract called Spirulysat^®^. Its composition of phycocyanins, polysaccharides, proteins, and vitamins makes it a valuable ingredient for premium nutraceutical formulations aimed at supporting antioxidant defences, immune function, and overall metabolic health [29,30,31].

Another significant research area is biofuel production, which frequently intersects with studies on bioremediation, wastewater treatment, adsorption, and heavy metal recovery. These applications reflect the dual potential of *Arthrospira* sp. as both a renewable energy source and an environmental remediation agent. The suitability of *Arthrospira* sp. for biofuel production is primarily attributed to its rapid growth rate, ability to thrive under alkaline and nutrient-rich conditions, and relatively high lipid and carbohydrate content, which can be converted into biodiesel, bioethanol, and other bioenergy carriers [32,33,34]. These features make it a promising candidate for sustainable, large-scale biofuel production while avoiding competition with conventional food crops for arable land.

In contrast, foliar applications are less frequently explored but are directly associated with plant bio-stimulation and biofertilizer use, indicating a growing interest in sustainable agriculture. In addition, *Arthrospira* sp. is a rich source of proteins, comprising up to 60–70% of its dry biomass. The protein profile of *Arthrospira* sp. not only supports its nutritional and functional food applications but also underpins its industrial relevance in cosmetics, dietary supplements, and pharmaceutical formulations. In the food and feed sector, *Arthrospira* sp. is primarily employed as a high-protein additive in poultry and aquaculture feed, enhancing nutritional profiles and promoting sustainable livestock and aquaculture practices [35].

**Figure 5:**
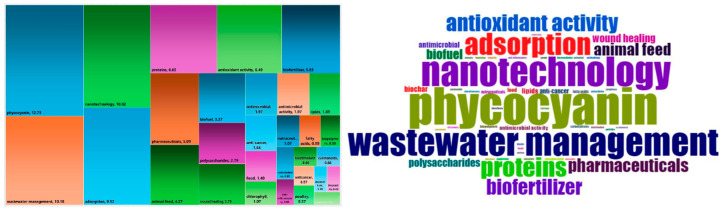
Tree map and word cloud of the different *Arthrospira* sp. applications based on popularity.

Another representation of frequently occurring keywords associated with *Arthrospira* sp. applications was generated using the Bibliometrix package in R (Figure 5). The obtained data illustrate the expanding industrial relevance of *Arthrospira* sp., spanning agriculture, sustainable materials, and personal care, and highlight its role as a multifunctional, bioactive, and environmentally friendly resource.

Consistently, phycocyanin emerged as one of the most frequently cited keywords in application-focused publications, followed by terms such as wastewater treatment, nanotechnology, and adsorption, highlighting the diversity of research directions.

Interestingly, more recent studies have expanded the range of *Arthrospira* sp. applications to include biofertilizers, bioplastic production, and cosmetic formulations. Interestingly, more recent studies have expanded the range of *Arthrospira* sp. applications to include biofertilizers, bioplastic production, and cosmetic formulations. The plant growth–promoting effects of *Arthrospira* sp. have garnered significant attention due to its richness in essential nutrients and bioactive compounds that enhance plant growth, nutrient uptake, and stress tolerance. In addition, the plant growth–promoting effects of *Arthrospira* sp. have garnered significant attention due to its richness in essential nutrients and bioactive compounds that enhance plant growth, nutrient uptake, and stress tolerance. In addition, it improves soil health and microbial activity while offering a sustainable and eco-friendly alternative to chemical fertilisers. These benefits have led to increased exploration of foliar applications for agricultural enhancement. It improves soil health and microbial activity while offering a sustainable and eco-friendly alternative to chemical fertilisers. These benefits have led to increased exploration of foliar applications for agricultural enhancement. For instance, the application of *Arthrospira* hydrolysate has been shown to significantly stimulate basil seedling growth, producing taller plants with greater biomass and larger leaf areas [36]. Similarly, the use of fermented *Spirulina maxima* as a biofertilizer improved rosemary growth, highlighting its potential as a natural and sustainable agricultural input [37].

With the growing market demand for sustainable materials, *Arthrospira* sp. has emerged as a promising source for bioplastics, aligning with the growing market demand for eco-friendly alternatives. Studies report that *Arthrospira platensis* can produce up to 7.8% (*w*/*w*) polyhydroxybutyrate (PHB) [38], while others have documented yields as high as 33% [39]. Moreover, the direct hot-pressing of whole *Spirulina* cells has been demonstrated to yield durable, cohesive bioplastics.

Moreover, the extensive bioactive properties of *Arthrospira* sp., particularly its antioxidant and anti-inflammatory activities, have also made it an attractive ingredient in cosmetic and skincare products. Recent reviews summarise advancements in incorporating *Arthrospira* sp. into formulations that target skin health, while experimental studies have demonstrated that cosmetic products containing *Spirulina* sp. can effectively protect against photoaging and enhance skin vitality [40,41].

#### 2.2.2. Dynamics of the Most Common Applications of *Arthrospira* sp.

The expanding and interdisciplinary nature of *Arthrospira* sp. research highlights its transition from traditional food and feed uses toward innovative applications in pharmaceuticals, environmental management, and emerging technologies.

**Figure 6:**
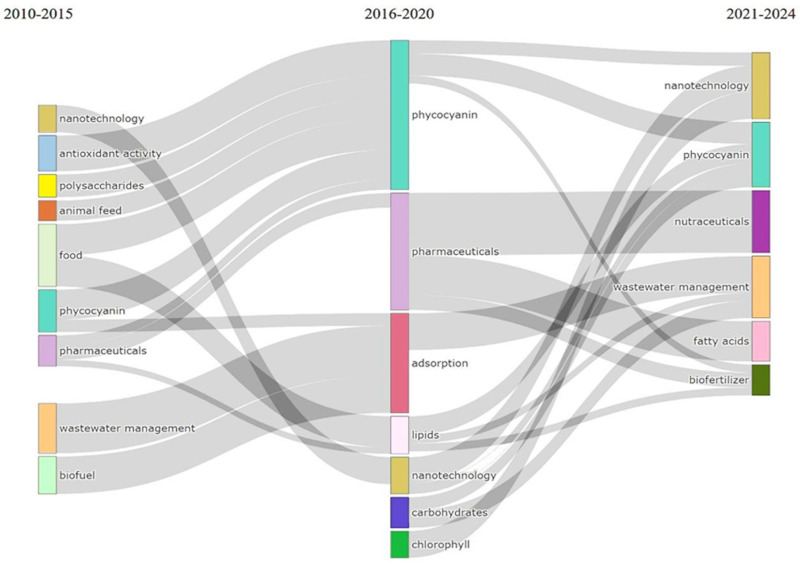
Thematic evolution map of key applications of *Arthrospira* sp. based on three-time intervals.

Among the diverse applications of *Arthrospira* sp., phycocyanin production, animal feed, adsorption, nanotechnology, and antioxidant-related uses have been the most established, exhibiting consistent and rapid growth over the years (Figure 6). While wastewater treatment applications emerged later, they have quickly become one of the fastest-growing research areas, highlighting *Arthrospira* potential in environmental management and industrial bioprocesses.

Despite *Arthrospira* sp.’s naturally high protein content, protein-focused applications only began to gain substantial research momentum after 2015, suggesting a delayed recognition of its nutritional and functional potential beyond traditional feed uses. Similarly, biofuel and biofertilizer applications remained relatively underexplored for an extended period; however, the past five years have seen a notable surge in interest, driven by global trends toward sustainable energy and eco-friendly agricultural solutions, resulting in increased visibility and research investment in these sectors.

Thematic evolution analysis (Figure 6) illustrates clear shifts in research priorities over time. Between 2010 and 2015, studies predominantly focused on human food, animal feed, wastewater management, and phycocyanin production, with pharmaceutical applications emerging at a smaller scale. In the subsequent period (2016–2020), research emphasis shifted markedly toward pharmaceutical applications and phycocyanin-related studies, while investigations into wastewater management increasingly highlighted *Arthrospira* sp.’s adsorption and bioremediation capabilities. Interestingly, during this phase, interest in its direct use as a human food source declined.

In the most recent period (2021–2024), research dynamics reveal further evolution: while studies on phycocyanin applications have slightly decreased, nanotechnology-based applications have demonstrated steady growth, reflecting the integration of *Arthrospira* sp. into advanced material science and biomedical research. Concurrently, biofertilizer applications have gained prominence, indicating a rising focus on sustainable agriculture and eco-friendly crop enhancement strategies. Collectively, these trends underscore the expanding and interdisciplinary nature of *Arthrospira* sp. research, transitioning from traditional food and feed uses toward innovative applications in pharmaceuticals, environmental management, and emerging technologies.

### 2.3. Commercial Production and Market Size of Arthrospira sp.

The commercial production of *Arthrospira* sp. has gained significant momentum in recent years due to the high economic value of its biomass and derived bioproducts [42]. Algal cultivation is widely regarded as one of the most sustainable production systems, relying on carbon dioxide, water, and essential macronutrients such as nitrogen and phosphorus while contributing to the principles of the circular bioeconomy. This approach not only supports carbon capture and nutrient recycling but also minimizes environmental impact compared to conventional agricultural and industrial processes.

Production of biomass and bioactive metabolites from *Arthrospira* involves several critical steps, beginning with cultivation under controlled conditions. *Arthrospira* sp. is typically grown in open raceway ponds or closed photobioreactors, with cultivation mode significantly influencing biomass yield, metabolite composition, and cellular physiology [25,43]. For instance, photobioreactors enable higher cell densities, precise control of light, pH, temperature, and nutrient supply, and reduce the risk of contamination, whereas open ponds are more cost-effective at large scale but offer lower productivity and a more variable metabolite profile [44]. Once the desired biomass density is achieved, harvesting is usually performed. A critical step in large-scale *Arthrospira* commercial production is efficient biomass harvesting, which plays a decisive role in determining overall process economics and product quality [45]. Harvesting of *Arthrospira* sp. is generally simpler than for many other microalgae due to its filamentous morphology and relatively large cell size, which allows efficient separation from the culture medium. Despite this simplicity, harvesting remains a quantitatively significant step in the overall production process, accounting for up to 30–40% of total production costs [25,46]. The choice of harvesting method not only affects economics but also directly influences the quality of the final product [47]. Gentle harvesting techniques preserve cell integrity and maintain high levels of bioactive compounds, including phycocyanin, gamma-linolenic acid, and proteins, which are essential for nutraceutical, functional food, and cosmetic applications [25,48]. Conversely, overly aggressive methods can damage cells, reducing pigment content, protein quality, and overall nutritional value. Therefore, optimizing harvesting strategies is critical to achieving a balance between biomass recovery efficiency, cost-effectiveness, and bioactive quality in commercial Spirulina production.

In practice, integrated harvesting strategies—combining two or more techniques such as filtration, flocculation, centrifugation, or sedimentation are often employed to balance efficiency and cost [49]. For commercial production of *Arthrospira* sp., primary flocculation or sedimentation is typically employed to recover biomass efficiently, while centrifugation may be applied only in specific high-value applications to further concentrate the biomass, balancing recovery efficiency with energy use and preservation of bioactive compounds [25,46,48]. Additionally, maintaining sterile and contamination-free conditions during harvesting is essential to ensure product safety, especially for food- and pharmaceutical-grade application [45]. Recycling the spent culture medium after appropriate treatment further enhances sustainability by conserving water and nutrients, ultimately lowering production costs and improving the environmental footprint of *Arthrospira* cultivation [47].

The harvested biomass can be further processed for nutritional or feed applications or further subjected to extraction processes to isolate bioactive metabolites. For example, phycocyanin, a blue pigment, is extracted from aqueous or buffer solutions and then purified by ammonium sulfate precipitation, ultrafiltration, and chromatography [48,50]. Highly purified phycocyanin is used in nutraceuticals, functional foods, and cosmetics due to its antioxidant, anti-inflammatory, and potential anticancer properties. Other compounds, such as carotenoids and gamma-linolenic acid (GLA), are obtained through solvent extraction or saponification, depending on the target metabolite [51,52]. These bioactive compounds are associated with cardiovascular health, anti-inflammatory effects, and immune support, and are incorporated into dietary supplements, fortified foods, and cosmeceuticals [53]. The yield, purity, and bioactivity of each metabolite depend on strain selection, cultivation conditions, and downstream processing, emphasising the importance of process optimisation for industrial and health-related applications [25,46].

Beyond traditional cultivation and extraction approaches, genetic engineering and Adaptive Laboratory Evolution (ALE) strategies have shown promise in enhancing biomass productivity, metabolite content, and stress tolerance of *Arthrospira* strains. Genetic manipulations can target pathways involved in phycocyanin, lipid, or carotenoid synthesis, while ALE can select for strains better adapted to high-density cultivation, variable light, or nutrient-limited conditions. For instance, a laboratory study reported that *Arthrospira* platensis cultures gradually acclimated to increasing NaCl concentrations (up to approximately 60 g L^−1^) were able to maintain biomass production comparable to that of conventional media, even under high-salinity conditions. However, their biochemical composition, including protein and phycocyanin content, was affected. These findings demonstrate the capacity of *Arthrospira* strains to adapt over time to salinity stress, effectively selecting for salt-tolerant phenotypes under prolonged exposure [54]. These strategies represent important avenues to further improve yields, resilience, and commercial feasibility, particularly in photobioreactor systems [55].

Global algae production has expanded rapidly, nearly doubling from 14.7 million tons to 30.4 million tons (wet weight) between 2005 and 2015 [56], a growth largely driven by macroalgae (seaweed) aquaculture. Accordingly, the global algae market, encompassing both macro- and microalgae, is projected to reach USD 6.3 billion by 2028, reflecting increasing demand for algae-based products in the food, nutraceutical, cosmetic, and pharmaceutical industries [57], Focusing specifically on microalgae, *Chlorella* and *Arthrospira* dominate the food supplement sector, collectively accounting for approximately 80% of the global microalgae food market [58]. Among these, *Arthrospira* species hold a leading market position due to their exceptional nutritional profile, functional properties, and documented health benefits [42]. According to Persistence Market Research, the global *Arthrospira* market was valued at approximately USD 619 million in 2024 and is expected to grow at a compound annual growth rate (CAGR) of 6.4%, reaching USD 955.5 million by 2031 [59]. Notably, the European market is projected to experience even stronger growth, with a CAGR of 13.73% between 2024 and 2031. Powdered products currently dominate the market, accounting for 71.3% of global *Arthrospira* sales, corresponding to a market value of USD 168.1 million.

Currently, North America leads the global *Arthrospira* market, holding 28.7% of the market share in 2022, with the United States alone accounting for more than 23%. In Europe, France, Germany, and Spain are the leading countries in commercial production of *Arthrospira* sp. biomass, with France contributing 65% of the region’s total production [60]. According to market forecasts, North America is expected to maintain its dominant position, with the United States experiencing the highest growth, followed by Canada. Additionally, Meticulous Research predicts that North America will continue to lead the *Arthrospira* market, accounting for 51.1% of the global share, with a market value of USD 327.2 million, followed by Asia–Pacific and Europe [61].

**Figure 7:**
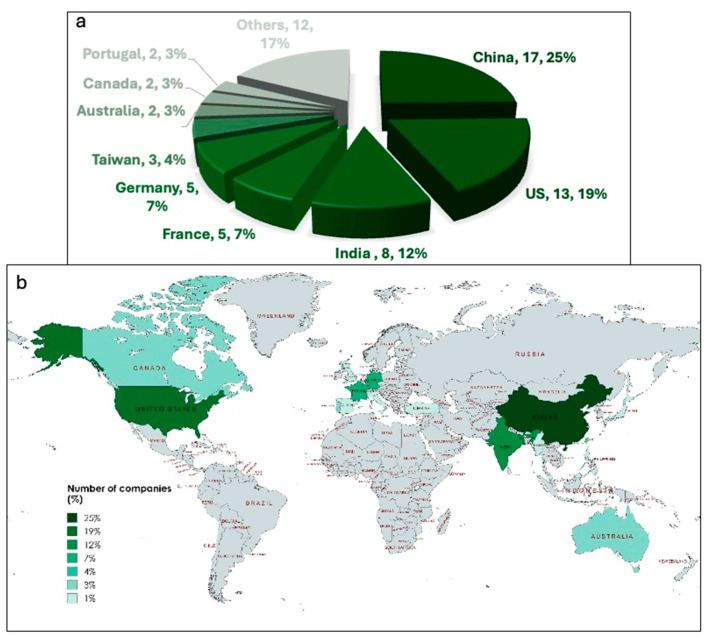
Global distribution of spirulina-producing companies: (**a**) The figure illustrates the international distribution of companies producing spirulina. The world map represents the geographic spread and relative concentration of companies by country, while the pie chart (**b**) presents the proportional contribution of major producing countries. China, the United States, and India account for the largest shares, followed by France, Germany, Taiwan, Australia, Canada, Portugal, and other countries, highlighting the worldwide development of the spirulina industry.

Similarly, Coherent Market Insights suggests that the Asia–Pacific region has strong potential to overtake other regions in the coming years due to rapid market expansion [62]. However, based on the number of registered companies producing *Arthrospira* (Figure 7), China currently leads with at least 17 registered commercial producers, followed by the United States and India. In Europe, France and Germany are the leading producers. With the increasing global focus on natural products, large-scale cultivation of *Arthrospira* is now primarily targeting the production of high-value proteins, particularly phycocyanin. This pigment–protein complex has applications in food, pharmaceuticals, and cosmetics, depending on its purity level [63]. By 2027, the global phycocyanin market is projected to reach USD 245.5 million [64]. The commercial price of phycocyanin varies significantly based on purity, ranging from USD 26 per milligram for partially purified phycocyanin to USD 208 per milligram for highly purified phycocyanin extract with a purity ratio of 3.5, which is suitable for nutraceutical, functional food, and cosmetic applications, whereas lower-purity extracts are primarily used as food colouring or bulk ingredients [65]. Consequently, advancements in the production, extraction, and purification of phycocyanin are key drivers enhancing the commercial value of *Arthrospira*, particularly in high-value markets where phycocyanin serves as a functional and bioactive ingredient.

Some of the largest *Arthrospira* production companies are listed inTable 1. However, obtaining precise data on the annual production rates of certain companies remains challenging.

### 2.4. Challenges Facing the Industry of Arthrospira sp.

#### 2.4.1. Safety Concerns

Due to their exceptional qualities, *Arthrospira* species are now commercially cultivated and consumed worldwide. However, experimental studies have demonstrated that modifications to the growth conditions of *Arthrospira* species can alter the strain’s quality [66]. If these changes are not properly controlled, they may negatively affect biomass quality. In fact, the safety of commercially available *Arthrospira* biomass has been a topic of debate. Food safety is a critical aspect of commercialisation, particularly because microalgae can be toxic either biogenically—producing phycotoxins—or non-biogenically by absorbing contaminants from their surroundings, such as heavy metals and residues [67].

The issue of toxicity is particularly challenging, as contamination can occur at any stage of production, from cultivation to packaging and storage [68]. For example, a study assessing the biomass quality of commercial Spirulina found significant heterogeneity in its nutritional composition, emphasising the need for continuous monitoring [69]. While the biomass did not exceed regulatory limits for heavy metals, pesticides, mycotoxins, and antibiotics, some samples contained elevated levels of polycyclic aromatic hydrocarbons.

Additionally, there have been reports of Spirulina consumption triggering anaphylaxis, potentially due to phycocyanin’s allergenic properties [70]. Another study confirmed similar findings, where Spirulina tablets caused allergic reactions, and skin prick tests yielded positive results [71]. Furthermore, testing commercial Spirulina products has revealed the presence of harmful microbial contaminants, including bacteria and fungi, as well as high concentrations of heavy metals—particularly lead—exceeding permissible levels [72]. Other studies have also detected elevated levels of lead and aluminium in Spirulina food supplements, exceeding recommended safety thresholds [73].

Beyond heavy metal contamination, research has shown that *Spirulina* food supplements contain allergenic proteins, such as the C-phycocyanin beta subunit, along with proteins resembling known food allergens. These include thioredoxins (maize allergen), superoxide dismutase (pistachio allergen), and triosephosphate isomerase (fish food allergen) [74]. Additionally, some Spirulina samples tested positive for microcystin and nodularin, specifically containing Microcystin-LR and Anatoxin-a [72]. Microcystins have also been detected in *Spirulina* fish food supplements [75], and products sold in Greece contained microcystin levels exceeding the tolerable daily intake for children and infants [76].

Interestingly, despite concerns about allergenicity, recent studies have explored *Arthrospira*’s potential in alleviating allergies, with evidence supporting its anti-allergic properties [77]. Ensuring the safety of commercially produced *Arthrospira* biomass requires a thorough assessment of its toxicity profile, including chemical composition, the presence of both biogenic and non-biogenic toxins compounds, and the quality of its protein [78].

#### 2.4.2. Regulatory Issues

Like any food product, *Arthrospira*-based products are subject to regulatory standards to ensure their safety as dietary supplements. These regulations include quality assessments at various production stages, focusing on the quantification of toxic metals, pathogens, and microcystins. In the United States, the FDA regulates *Arthrospira* products and has classified them as Generally Recognised as Safe (GRAS) at a daily dosage of 3–6 g [79]. Despite such regulations, a key biosafety concern remains the variability in quality standards between different batches of the same product, highlighting the need for optimised assessment protocols for *Arthrospira*-based products [69].

In the European Union, foods without a history of significant consumption before 1997 must undergo safety assessment under the Novel Foods Regulation (EC) No. 258/97, now replaced by Regulation (EU) 2015/2283, before being authorized for commercialization. However, since *Arthrospira* was consumed in Europe well before 1997, its marketing does not require a safety assessment [80]. Consequently, importing *Arthrospira*-based products from non-EU countries into Europe only necessitates an international phytosanitary certificate, which verifies the product’s origin, traceability, and freedom from pests [81]. However, this certification does not always account for the quality of algae-based products, as European regulations lack specific risk assessment criteria for microalgae-derived products [82].

In addition to safety, clinical and preclinical studies have demonstrated the therapeutic potential of *Arthrospira* sp. (*Spirulina*) across multiple health applications worldwide. For cardiovascular and lipid-lowering effects, trials in India and Iran used 1–8 g/day of Spirulina powder for 6–12 weeks, resulting in significant reductions in LDL cholesterol and triglycerides [83]. Antioxidant and immune-modulatory benefits were observed with 2–4.5 g/day for 12 weeks in Korea and Mexico, improving antioxidant status and immune function [84]. Glucose-lowering effects were reported with 2–8 g/day for 12–24 weeks in Brazil and Thailand, reducing markers of inflammation and improving glycemic control [85].

## 3. Conclusions

The commercial production of *Arthrospira* sp. represents one of the most successful examples of sustainable algal biotechnology, combining environmental benefits with high economic value. Globally, *Arthrospira* continues to attract scientific and commercial interest due to its exceptional nutritional composition, versatile biochemical profile, and remarkable resilience under diverse and often extreme environmental conditions. The bibliometric analysis presented in this work highlights a dynamic research evolution—shifting from traditional food and feed applications toward value-added bioproducts such as phycocyanin, bioactive compounds, bioplastics, nanomaterials, and biofertilizers. This transition reflects the growing demand for sustainable, multifunctional, and naturally derived ingredients across the health, cosmetic, pharmaceutical, and agricultural industries.

Despite major advances, large-scale production of *Arthrospira* still faces challenges related to cost, scalability, and environmental sustainability. High operational expenses associated with nutrient supply, harvesting, and drying remain key economic bottlenecks. Nutrient input, particularly nitrogen and phosphorus, accounts for the largest share of costs, highlighting the need for efficient resource management. Additionally, land use and energy requirements, especially for aeration, mixing, temperature regulation, and lighting, pose further constraints, emphasising the importance of renewable energy integration and sustainable site selection. In summary, while remarkable progress has been made toward industrialising *Arthrospira* production, achieving truly large-scale, cost-effective, and sustainable systems requires continued innovation across cultivation design, process integration, and resource management. Through the convergence of biotechnology, engineering, and sustainability principles, *Arthrospira* can become not only a valuable superfood but also a cornerstone of the circular bioeconomy, supporting food security, environmental restoration, and global bio-based development.

### Future Perspectives

Future research should prioritise the development of integrated biorefinery frameworks to maximise the valorisation of *Arthrospira* biomass across multiple product pathways. Cascading extraction strategies, recovering high-value pigments such as phycocyanin first, followed by proteins, lipids, and polysaccharides, can markedly increase economic efficiency while ensuring near-zero-waste utilisation, fully aligning with circular bioeconomy principles [86,87].

Advances in strain selection and metabolic engineering are essential to develop robust *Arthrospira* strains capable of withstanding fluctuations in light, salinity, and temperature, enabling efficient outdoor cultivation in diverse environments, including arid and saline regions. Integrating automation, digital monitoring, and AI-based optimisation can further enhance productivity and consistency in large-scale production.

Sustainability assessment tools, including techno-economic analysis (TEA) and life cycle assessment (LCA), have become increasingly important for evaluating the industrial feasibility and environmental sustainability of *Arthrospira* sp. production systems and should be systematically embedded in future cultivation and biorefinery developments. TEA studies indicate that production costs are primarily driven by cultivation mode (open ponds versus photobioreactors), energy demand for mixing and harvesting, nutrient inputs, and downstream processing steps, particularly drying and metabolite purification [86]. The co-production of high-value compounds, such as phycocyanin and other bioactive extracts, has been shown to significantly enhance economic viability by offsetting biomass production costs. From an environmental perspective, LCA studies generally report that *Arthrospira* cultivation offers a favourable protein yield per hectare and a lower land footprint compared to conventional crops; however, environmental impacts can increase substantially when energy-intensive operations and synthetic fertilisers are used [86,88]. In this context, the use of renewable energy sources, alternative water and nutrient inputs such as wastewater streams, and integrated biorefinery approaches have been identified as key strategies to reduce greenhouse gas emissions, water consumption, and overall environmental burden while enabling nutrient recovery and environmental remediation [21]. Despite growing interest, TEA and LCA studies on *Arthrospira* remain relatively limited and often rely on heterogeneous assumptions, underscoring the need for harmonised methodologies and region-specific assessments. Overall, integrating TEA–LCA frameworks into future *Arthrospira* research is essential to guide process optimisation, support scale-up and policy decisions, and ensure that commercial development aligns with long-term sustainability and circular bioeconomy objectives.

Overall, combining technological innovation with sustainable practices will consolidate *Arthrospira*’s role as a versatile bioresource for nutrition, health, biotechnology, and environmental applications, supporting the global transition toward a resilient, circular, and bio-based economy.

## 4. Materials and Methods

### 4.1. Study Design

**Figure 8:**
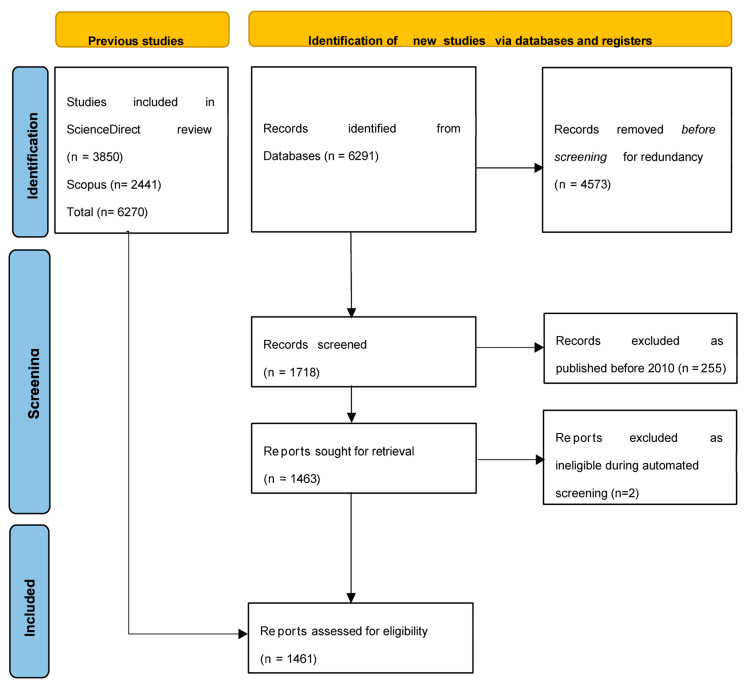
Study flowchart (PRISMA flowchart).

This systematic review was conducted in strict accordance with the PRISMA 2020 (Preferred Reporting Items for Systematic Reviews and Meta-Analyses) guidelines [89]. All documents published between January 2010 and October 2024 in Scopus- indexed journals were retrieved and included in the present bibliometric analysis. Scopus and ScienceDirect were selected as the primary data sources due to their extensive coverage of peer-reviewed literature across diverse disciplines, including science, technology, and applied research, as well as their advanced functionalities for data export and citation tracking. Figure 8 illustrates the study selection process using the PRISMA flow diagram.

The search strategy was designed to collect studies related to *Arthrospira*, Spirulina, and *Halospirulina*, with a specific focus on their applications. The following Boolean query was used in the Scopus search field:

TITLE-ABS-KEY “*Arthrospira*” OR TITLE-ABS-KEY “*Spirulina*” OR TITLE-ABS-KEY “Halospirulina” AND TITLE-ABS-KEY “application” AND PUBYEAR > 2010 AND PUBYEAR < 2024

The search was limited to English-language resources, including journal articles, reviews, conference papers, and book chapters, to ensure data consistency and reproducibility. The initial search resulted in 1463 documents, which were subsequently exported in CSV format containing complete bibliographic information, including authors, titles, abstracts, keywords, sources, affiliations, and citation metrics (Figure 1).

### 4.2. Data Preprocessing

Before analysis, the bibliographic dataset was carefully screened to remove duplicate entries, incomplete records, and non-relevant publications (e.g., those not focusing on Spirulina or its related genera). The refined dataset was then standardised to correct inconsistencies in author names, country affiliations, and keyword variations (for example, *Arthrospira* platensis vs. *Spirulina* platensis).

To ensure accurate mapping of publication trends and co-occurrence relationships across the dataset, redundant records were eliminated, and harmonization procedures were applied to unify recurring terms and metadata fields.

### 4.3. Data Analysis

The cleaned and non-redundant bibliographic data were imported into RStudio (version 4.3.3) and analysed using the Bibliometrix package [90]. This comprehensive bibliometric tool enabled the extraction and computation of various performance and collaboration indicators, including: (i) Document type distribution (e.g., articles, reviews, conference papers); (ii) Annual scientific production and citation trends; (iii) Most productive journals and publication sources; (iv) Authorship and institutional collaboration networks; (v) Geographical distribution of publications by country; and (vi) Keyword frequency and thematic evolution.

The analysis outputs were visualised using R’s built-in plotting functions and the Biblioshiny web interface, allowing the generation of publication trend graphs, bar charts, and collaboration maps that illustrate the structural dynamics of Spirulina research over time.

### 4.4. Visualisation and Mapping

To complement the R-based analysis, a co-occurrence network map of keywords and terms appearing in titles, abstracts, and author keywords was constructed using VOSviewer (version 1.6.20). This software was employed to visualise relationships among frequently occurring terms, offering insights into the conceptual structure and major research hotspots within the field.

A minimum occurrence threshold of 20 was applied to filter and retain only the most significant terms, thereby improving the readability and interpretability of the resulting network. The visualisation revealed several clusters representing key thematic domains (e.g., biotechnological applications, nutritional value, and environmental uses of Spirulina), with nodes indicating keywords and links representing co-occurrence strength.

### 4.5. Complementary Analyses and Data Representation

Descriptive representations of data, including document type percentages, country-wise publication shares, and journal contribution ratios, were performed using Microsoft Excel. Pie charts, tables, and summary figures were generated to provide a clear overview of publication characteristics. These visualisations complemented the statistical and network analyses, offering a comprehensive understanding of global research dynamics on Spirulina and its related genera.

## Figures and Tables

**Table 1:** Companies producing *Arthrospira* sp. commercially.

Company	Country	Annual Production	Reference
Yunnan Green A Biological Project Co., Ltd.	China	3000 metrics ton of *Spirulina*, *Chlorella*, and *Haematococcus Pluvialis*	(TOP 10 COMPANIES IN SPIRULINA MARKET, https://www.marketdataforecast.com/market-reports/spirulina-market accessed on 4 January 2025).
Inner Mongolia Rejuve Biotech Co. Ltd.	China	*Spirulina* powder is 1100 tons per year and Spirulina tablets are 100 tons per year.	(TOP 10 COMPANIES IN SPIRULINA MARKET https://www.marketdataforecast.com/market-reports/spirulina-market, accessed on 4 January 2025).
DIC Corporation	Japan	1000 tons per year Revenue of $5.42 Billion	(https://www.dic-global.com/en/contents/scene/spirulina accessed on 4 January 2025).
Fuqing King Dnarmsa Spirulina Co. Ltd.	China	1600 ton of Spirulina	(TOP 10 COMPANIES IN SPIRULINA MARKET https://www.marketdataforecast.com/market-reports/spirulina-market accessed on 4 January 2025).
Clos Sainte Aurore	France	NA	(https://www.verifiedmarketreports.com/blog/top-spirulina-companies/ accessed on 6 January 2025).
Spirulina Viva	Mexico	NA	(https://www.verifiedmarketreports.com/blog/top-spirulina-companies/ accessed on 6 January 2025).
SPIFORM	France	NA	(https://www.verifiedmarketreports.com/blog/top-spirulina-companies/ accessed on 6 January 2025).
Spirulina La Capitelle	France	NA	(https://www.verifiedmarketreports.com/blog/top-spirulina-companies/ accessed on 6 January 2025).
Aurospirul	India	NA	(https://www.verifiedmarketreports.com/blog/top-spirulina-companies/ accessed on 6 January 2025).
Far East Microalgae Industries, Co., Ltd.	Taiwan	NA	(https://www.verifiedmarketreports.com/blog/top-spirulina-companies/ accessed on 6 January 2025).
Givaudan SA	Switzerland	NA	(Europe Spirulina Market by Size, Share, Forecasts, & Trends Analysis (https://www.meticulousresearch.com/product/europe-spirulina-market-5499 accessed on 4 January 2025)).
Cyanotech Corporation	U.S.	Revenue of $23.071 Million.	(IMIR Market Research Pvt. Ltd. Market research reports, consulting (https://www.intellectualmarketinsights.com/blogs/top-10-companies-in-the-spirulina-market-by-imir-market-research accessed on 4 January 2025)).
Aliga Microalgae	Denmark	NA	(Europe Spirulina Market by Size, Share, Forecasts, & Trends Analysis (https://www.meticulousresearch.com/product/europe-spirulina-market-5499 accessed on 4 January 2025)).
Roquette Klötze GmbH & Co. KG	Germany	NA	(Europe Spirulina Market by Size, Share, Forecasts, & Trends Analysis (https://www.meticulousresearch.com/product/europe-spirulina-market-5499 accessed on 4 January 2025)).
ALGALIMENTO SL	Spain	NA	(Europe Spirulina Market by Size, Share, Forecasts, & Trends Analysis (https://www.meticulousresearch.com/product/europe-spirulina-market-5499 accessed on 4 January 2025)).
Sea & Sun Organic GmbH	Germany	NA	(Europe Spirulina Market by Size, Share, Forecasts, & Trends Analysis (https://www.meticulousresearch.com/product/europe-spirulina-market-5499 accessed on 4 January 2025)).
BlueBioTech Group	Germany	NA	(Europe Spirulina Market by Size, Share, Forecasts, & Trends Analysis (https://www.meticulousresearch.com/product/europe-spirulina-market-5499 accessed on 4 January 2025)).
E.I.D.—Parry Limited	India	NA	(Europe Spirulina Market by Size, Share, Forecasts, & Trends Analysis (https://www.meticulousresearch.com/product/europe-spirulina-market-5499 accessed on 4 January 2025)).
June Pharmaceutical Company Ltd.	Myanmar	NA	
Australian Spirulina	Australia	Revenue of $215.2 Million.	(IMIR Market Research Pvt. Ltd. Market research reports, consulting (https://www.intellectualmarketinsights.com/blogs/top-10-companies-in-the-spirulina-market-by-imir-market-research accessed on 4 January 2025)).

## Data Availability

No new data were created or analyzed in this study.

## Abbreviations

The following abbreviations are used in this manuscript:

CAGR	Compound Annual Growth Rate
GRAS	Generally Recognized as Safe
LCA	life cycle assessment
TEA	Techno-economic analysis
WHO	World Health Organization
USFDA	United States Food Drug Administration
